# Adaptive Conformal Cooling of Injection Molds Using Additively Manufactured TPMS Structures

**DOI:** 10.3390/polym14010181

**Published:** 2022-01-03

**Authors:** Seo-Hyeon Oh, Jong-Wook Ha, Keun Park

**Affiliations:** Department of Mechanical System Design Engineering, Seoul National University of Science and Technology, Seoul 01811, Korea; osh516406@seoultech.ac.kr (S.-H.O.); 16100233@seoultech.ac.kr (J.-W.H.)

**Keywords:** injection molding, conformal cooling channel, additive manufacturing, micro-cellular structure, triple periodic minimal surface

## Abstract

In injection molding, cooling channels are usually manufactured with a straight shape, and thus have low cooling efficiency for a curved mold. Recently, additive manufacturing (AM) was used to fabricate conformal cooling channels that could maintain a consistent distance from the curved surface of the mold. Because this conformal cooling channel was designed to obtain a uniform temperature on the mold surface, it could not efficiently cool locally heated regions (hot spots). This study developed an adaptive conformal cooling method that supports *localized-yet-uniform* cooling for the heated region by employing micro-cellular cooling structures instead of the typical cooling channels. An injection molding simulation was conducted to predict the locally heated region, and a mold core was designed to include a triply periodic minimal surface (TPMS) structure near the heated region. Two biomimetic TPMS structures, Schwarz-diamond and gyroid structures, were designed and fabricated using a digital light processing (DLP)-type polymer AM process. Various design parameters of the TPMS structures, the TPMS shapes and base coordinates, were investigated in terms of the conformal cooling performance. The mold core with the best TPMS design was fabricated using a powder-bed fusion (PBF)-type metal AM process, and injection molding experiments were conducted using the additively manufactured mold core. The developed mold with TPMS cooling achieved a 15 s cooling time to satisfy the dimensional tolerance, which corresponds to a 40% reduction in comparison with that of the conventional cooling (25 s).

## 1. Introduction

Injection molding is the most widely-used polymer processing technology, in which a hot polymer melt fills the mold cavity and is solidified by subsequent coolant cooling. In injection molding, the mold temperature must be kept high to improve the fluidity of the polymer melt and the relevant part quality, while it must be kept low below the softening temperature of the polymer for appropriate demolding [1]. Mold cooling is an essential process in injection molding, in which a coolant circulates inside the mold through a series of cooling channels. A cooling channel is generally fabricated inside the mold by a machining (i.e., drilling) process, and thus a cooling circuit consists of a series of straight cooling channels [2]. These straight cooling channels, however, result in non-uniform cooling for a curved shape because the distance between the mold surface and cooling channel is not uniform. This non-uniform cooling has disadvantages in injection molding, including a delay in cooling time and part warpage due to non-uniform shrinkage [3].

To overcome these disadvantages, additive manufacturing (AM) has been used to fabricate conformal cooling channels inside injection molds [4]. The conformal cooling channel was usually designed to maintain a consistent distance from the curved mold surface, and the resulting complicated shape was fabricated utilizing the high design flexibility of the AM technology [5]. Various studies have used metal AM processes such as powder bed fusion (PBF) or directed energy deposition (DED) to develop injection molds containing conformal cooling channels [6,7,8,9,10,11]. This conformal cooling channel with a consistent distance from the mold surface was based on the assumption that the initial temperature of the mold surface was uniform. However, this assumption might be incorrect because a molded part may have locally heated regions, called *hot spots* [12]. Therefore, the conventional channel-type conformal cooling under the rule of the consistent distance cannot efficiently cool the locally heated regions. Moreover, conventional channel-type conformal cooling has a limitation in localized cooling because all channels should be in the form of a connected circuit [13,14,15]. Other disadvantages of an additively manufactured injection mold are the high manufacturing costs and low surface quality. To overcome these disadvantages, several studies developed hybrid molds for conformal cooling by combining the traditional machining and AM processes [16,17].

This study developed a novel mold design for localized conformal cooling with adaptive temperature control by employing micro-cellular cooling structures instead of the typical conformal cooling channels. Micro-cellular structures are defined by their repeated cell structures of lattice-type or surface-type unit cells [18], which can also be fabricated by utilizing the high design complexity of AM [19,20,21]. The cellular structures have mainly been used to design lightweight structures by enhancing structural efficiency [22,23,24]. They have also been used to control heat transfer characteristics by differentiating thermal conductivity [25,26,27]. While the previous studies mainly used lattice-type cellular structures, surface-type cellular structures were also used to improve the structural or thermal efficiency. The most popular surface-type cellular structure is a triply periodic minimal surface (TPMS), which was inspired by biological organisms [28]. TPMSs provide various types of continuous and smooth shell structures with large surface areas and continuous internal channels, and thus have been used in thermo-fluidic applications such as fluid mixers or heat exchangers [29,30,31].

In this study, an adaptive conformal cooling method was proposed by embedding a TPMS cooling structure inside a mold core, instead of using conventional channel type conformal cooling. Numerical simulation of injection molding was conducted to predict hot spot locations in which a TPMS cooling structure was placed. Two biomimetic TPMS structures, Schwarz diamond and gyroid structures, were designed to be embedded near hot spot regions. Various design parameters, the shape and volume fraction of the TPMS, were investigated in terms of the cooling performance. To compare the cooling performance of various TPMS designs, polymer AM was used to fabricate the corresponding TPMS, and the equivalent mold heating experiments were conducted by circulating hot water through the additively manufactured polymer TPMS inserts. The best TPMS design was then selected by comparing the results of the mold heating experiments, which enabled *localized-yet-uniform* temperature distribution near the hot spots. The mold core including the best TPMS design was fabricated using the PBF-type metal AM process. Injection molding experiments were then conducted using the fabricated mold. The cooling performance of the developed TPMS mold was compared with that of typical channel-type cooling by analyzing the dimensional accuracy of the molded parts according to cooling time.

## 2. Materials and Methods

### 2.1. Materials

For injection molding, polypropylene (PP, H1500, LG Chemical Co., Seoul, Korea) was used as a molding material. The density, melting temperature, and melt flow index were 0.9 g/cm^3^, 150 °C, and 1.2 g/min, respectively. A high-strength aluminum alloy (AA7075, Dongyang Aluminum Co. Ltd., Daejon. Korea) was used as a mold core, while the other mold plates were fabricated using AISI-1055 steel. Table 1 compares the thermal and mechanical properties of these materials.

For polymer AM, a photo-curable acrylic resin (3DK83I, Carima Inc., Seoul, Korea) was used. This photopolymer was developed for a digital light processing (DLP) type 3D printer and was optimized to be cured by the UV irradiation at 405 nm wavelength. The density and viscosity of this photopolymer was 1.10 g/cm^3^ and 365 cps, respectively. For metal AM, aluminum alloy powders (AlSi7Mg, Tekna Advanced Materials Inc., Sherbrooke, QC, Canada) were used because their mechanical and thermal properties are similar to those of AA7075, as compared in Table 1.

### 2.2. Design of the Injection Mold

**Figure 1:**
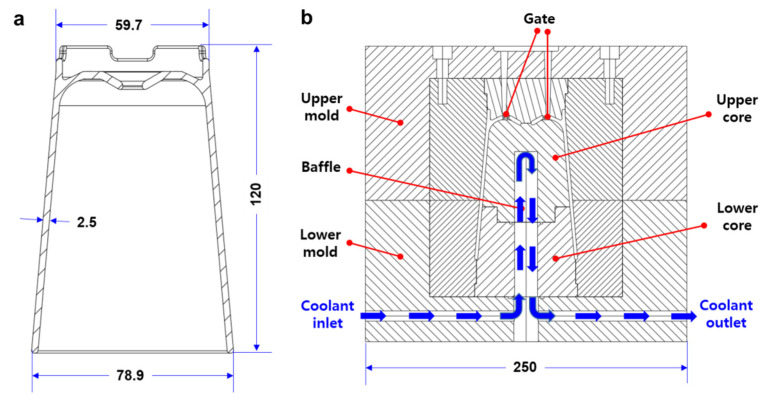
Design of an injection mold for a cup-shaped part: (**a**) Sectional view and dimensions of the cup-shaped part (unit: mm). (**b**) Design of the injection mold with baffle cooling.

An injection mold was designed for a cup-shaped part. Figure 1a shows the sectional view and dimensions of the cup-shaped part, of which the upper and lower diameters and height and thickness were 59.7, 78.9, 120, and 2.5 mm, respectively. This part was designed for use in a sanitary gargle with drain function. Figure 1b shows the design configuration of the injection mold. The mold was designed to have the upper and lower parts, and two separated mold cores (i.e., the upper and lower cores) were included in the lower mold. For appropriate cooling of the core, a baffle was inserted in the core through which the coolant circulated, as shown in Figure 1b. In this mold design, all mold components were fabricated by the machining process, while the upper core was also fabricated by an additively manufactured TPMS cooling structure for improved cooling capability.

### 2.3. Design of TPMS Structures

In this study, TPMS structures were used to develop micro-cellular pore structures for conformal cooling. TPMSs provide smooth shell structures with large surface areas and continuous internal channels, and thus increase heat transfer efficiency with relatively low pressure drop.

We used two types of TPMS structures, gyroid (G-surface) and Schwartz diamond (D-surface). In the Cartesian coordinate, implicit forms of G- and D-surfaces are represented by Equations (1) and (2), respectively.

(1) $$\;\;\;\;\varphi_{G}^{1}=\sin\left(x\right)\cos\left(y\right)+\sin\left(y\right)\cos\left(z\right)+\sin\left(z\right)\cos\left(x\right)-C_{G}^{1}=0$$

(2) $$\begin{array}{ll} \varphi_{D}^{1} & =\sin\left(x\right)\sin\left(y\right)\sin\left(z\right)+\sin\left(x\right)\cos\left(y\right)\cos\left(z\right)\\ & +\cos\left(x\right)\sin\left(y\right)\cos\left(z\right)+\cos\left(x\right)\cos\left(y\right)\sin\left(z\right)-C_{D}^{1}=0 \end{array}$$

where *φ* is the implicit function for a TPMS surface, and *C* is a constant that determines the surface thickness. The superscript *1* means that the mathematical expressions are based on the Cartesian coordinate (*x*, *y*, *z*). The subscripts G and D indicate the gyroid and diamond surfaces, respectively. These equations can also be expressed based on the cylindrical coordinate (*r*, *θ*, *z*), as expressed in Equations (3) and (4).

(3) $$\varphi_{G}^{2}=\sin\left(r\right)\cos\left(\theta \right)+\sin\left(\theta \right)\cos\left(z\right)+\sin\left(z\right)\cos\left(r\right)-C_{G}^{2}=0$$

(4) $$\begin{array}{ll} \varphi_{D}^{2} & =\sin\left(r\right)\sin\left(\theta \right)\sin\left(z\right)+\sin\left(r\right)\cos\left(\theta \right)\cos\left(z\right)\;\\ & +\cos\left(r\right)\sin\left(\theta \right)\cos\left(z\right)+\cos\left(r\right)\cos\left(\theta \right)\sin\left(z\right)-C_{D}^{2}=0 \end{array}$$

where the superscript *2* means that the mathematical expressions are based on the cylindrical coordinate.

**Figure 2:**
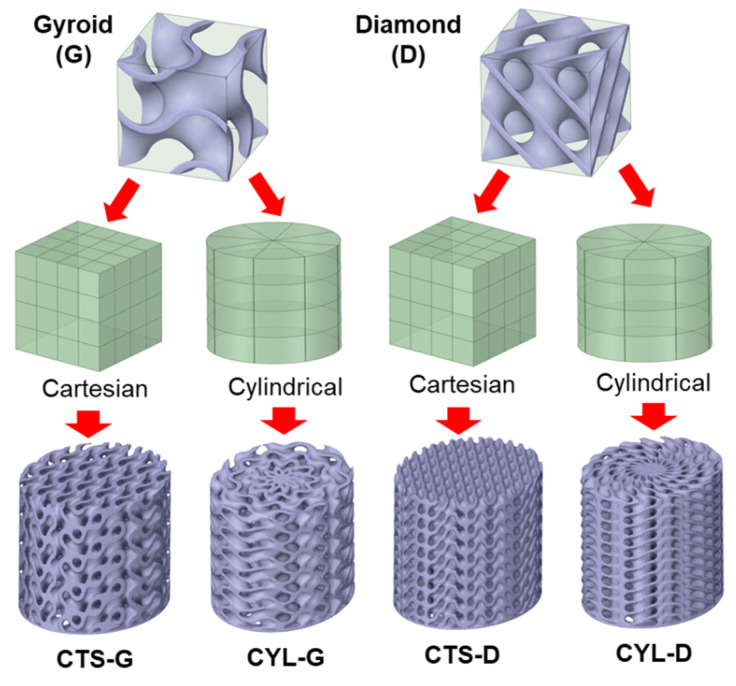
Design of TPMS structures with different combinations of unit cells and base coordinates.

For their use as a conformal cooling component of a mold core, these TPMS surfaces were generated based on an elliptic cylinder. The elliptic cylinder was designed with a major diameter of 38.4 mm, minor diameter of 32.4 mm, and height of 35 mm. Combinations of different TPMS cells (i.e., G- or D-surface) and base coordinates were then applied to design four TPMS structures, as shown in Figure 2. These four TPMS designs were named CTS-G, CYL-G, CTS-D, and CYL-D, according to the TPMS shape and coordinate type. For all TPMS designs, the target volume fraction was set to 50%, and the resulting volume fractions were within 50 ± 1%, as listed in Table 2. The corresponding surface areas are also compared in Table 2, which indicates that the diamond TPMS ensures a larger surface area than the gyroid TPMS.

### 2.4. Additive Manufacturing

To fabricate TPMS cooling structures, polymer and metal AM processes were used. The polymer AM was used for the mold heating test, in which various designs of TPMS structures were fabricated and inserted in the test section. A digital light processing (DLP) type 3D printer (IM96, Carima Inc., Seoul, Korea), was used in the polymer AM. This printer uses a digital micromirror device (DMD) chip with high definition (1920 × 1080), and the resulting pixel size is 50 μm. A UV lamp with a 405 nm wavelength was used, and the relevant layer thickness and irradiation time per layer were set to 100 μm and 3.1 s, respectively. The printed samples were additionally cured using a UV-curing machine (CL300, Carima Inc., Seoul, Korea) for 30 s under 405 nm UV irradiation.

After the mold heating test, the best TPMS design was selected and fabricated using metal AM, in which a mold core for injection molding experiments was additively manufactured. A powder bed type 3D printer (CL M2 Cusing, GE Additive Inc., Cincinnati, OH, USA) and AlSi7Mg powders were used for the metal AM. The laser power and speed were set to 370 W and 1600 mm/s. The layer thickness was set to 40 μm, and the beam spot diameter and hatching distance were set to 110 and 112 μm, respectively. After the printed parts were separated from the build plate, additional heat treatment was conducted for 90 min at a temperature of 280 °C. Post-processing was then performed to increase the surface finish and dimensional accuracy of the additively manufactured core part.

### 2.5. Numerical Simulation for Injection Molding

**Figure 3:**
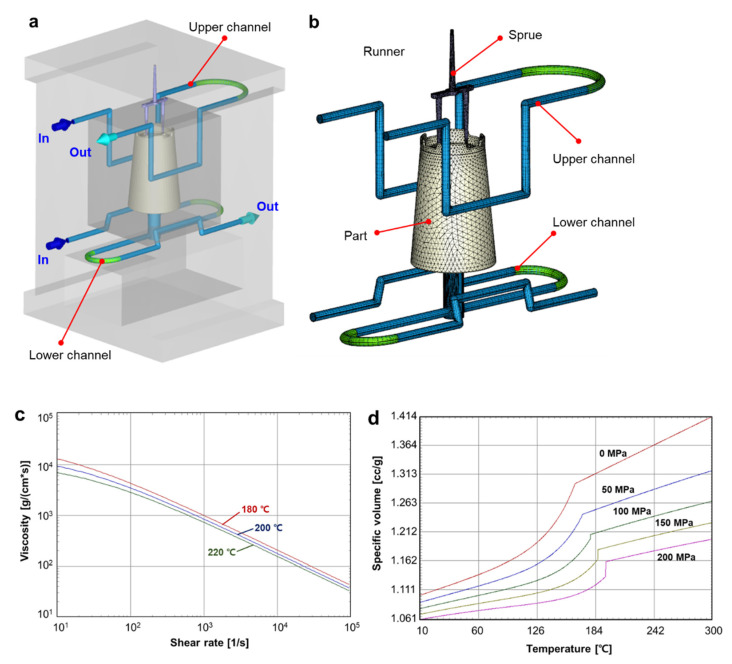
Description of the injection molding simulation conditions: (**a**) Configuration of the simulation model. (**b**) Finite element mesh. (**c**) Viscosity chart of the PP resin. (**d**) PvT diagram of the PP resin (H1500, LG Chemical).

To investigate the cooling performance of the designed cooling structures, we conducted a numerical simulation for the injection molding process. The injection molding simulation was performed for the filling, packing, and cooling stages. Moldex3D^®^ (CoreTech System Co. Ltd., Zhubei, Taiwan) was used in the injection molding simulation. Figure 3a shows the simulation model, including the sprue, runner, and upper and lower cooling channels. The boundary conditions, including the inlets and outlet of the cooling channels, are also marked in Figure 3a. This simulation model was discretized with the solid tetrahedral and hexahedral mesh, as demonstrated in Figure 3b. A total of 530,269 elements were used to construct the simulation model. The injection molding conditions are given in Table 3.

For the simulation of the mold filling process, the viscosity of the polymer melt (*η*) is expressed using the five-constant cross-exp model, as given in the following equation:

(5) $$\eta \left(T,\;\dot{\gamma },p\right)=\frac{\eta_{0}\left(T,\;p\right)}{1+{\left[\frac{\eta_{0}\dot{\gamma }}{\tau^{*}}\right]}^{1-n}}$$

where *n* and τ^*^ are the power-law constant and transition stress, respectively. *η*_0_ is a zero shear rate viscosity expressed by a WLF-type model [32].

(6) $$\eta_{0}\left(T,\;p\right)=D_{1}exp\left[-\frac{A_{1}\left(T-T^{*}\right)}{A_{2}+\left(T-T^{*}\right)}\right]$$

where *A*_1_ and *A*_2_ are material constants, and T^*^ is the glass transition temperature, which is expressed as a function of pressure [33].

(7) $$T^{*}=D_{2}+D_{3}p$$

For the simulation of the packing process, the specific volume (*ν*) is expressed using the two-domain Tait equation, as given in the following equation.

(8) $$\nu \left(T,\;p\right)=\nu_{0}\left(T\right)\left[1-C\ln\left(1+\frac{p}{B\left(T\right)}\right)\right]$$

where *ν*_0_(T) and *B*(T) are given as the following equations.

(9) $$\nu_{0}\left(T\right)=\{\begin{array}{c} \;b_{1,l}+b_{2,l}\bar{T},\;for\;T>T^{*}\\ \;b_{1,s}+b_{2,s}\bar{T},\;for\;T<T^{*} \end{array}$$

(10) $$B\left(T\right)=\{\begin{array}{c} \;b_{3,l}\;\exp\left(-b_{4,l}\bar{T}\right),\;for\;T>T^{*}\\ \;b_{3,s}\;\exp\left(-b_{4,s}\bar{T}\right),\;for\;T<T^{*} \end{array}$$

where *b*_1_ through *b*_4_ are material parameters, and the subscripts *l* and *s* denote the liquid and solid states, respectively. $\bar{T}$ is the effective temperature, which is expressed by the following equations.

(11) $$\bar{T}=T-b_{5}$$

where *b*_5_ is a material constant. The viscosity and PvT diagrams of the PP resin (H1500, LG Chemical) are shown in Figure 3c,d, respectively.

### 2.6. Mold Heating Tests

**Figure 4:**
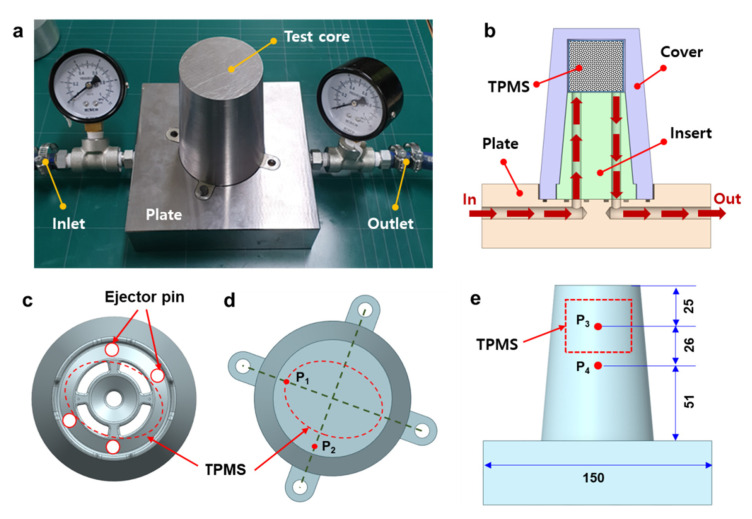
Description of the mold heating test: (**a**) Experimental setup. (**b**) Sectional configuration of the test mold. (**c**) Position of the ejector pins and the TPMS cooling structures. (**d**) Sample measurement points on the top surface (P_1_ and P_2_). (**e**) Sample measurement points on the side surface (P_3_ and P_4_).

To evaluate the cooling performance of the various TPMS cooling structures, equivalent mold heating tests were conducted. Figure 4a shows the experimental setup for the mold heating test. A test mold was fabricated by assembling a mold core into a base plate. The mold core consists of a cover, an insert block, and a TPMS structure, as shown in Figure 4b. Hot water with a temperature of 80 °C was injected into the mold using a hot water supply (HF-40, Hyundai FA, Seoul, Korea). The hot water reaches the core through the cooling channel and passes through the TPMS structure. Accordingly, the upper section of the mold core needs to be heated intensively. This heating test was designed to simulate the cooling stage of injection molding and to infer the cooling capability in the actual injection molding process by considering the equivalent heating capability.

Figure 4c shows a top view of the molded part, including the outer profile of the TPMS structure and the ejector pin locations. Here, the elliptic shape of the TPMS was designed to avoid interference with the ejector pins. Figure 4d shows a top view of the test mold core, including two sample point locations for temperature measurements, P_1_ and P_2_. Figure 4e shows a side view of the test mold, including two sample points, P_3_ and P_4_. An infrared thermal imaging system (FLIR E50, FLIR Systems Inc., Wilsonville, OR, USA) was used to measure the temperature distribution of the mold core. Here, the mold surface was coated with black graphite spray for measurement accuracy.

### 2.7. Injection Molding Experiments

**Figure 5:**
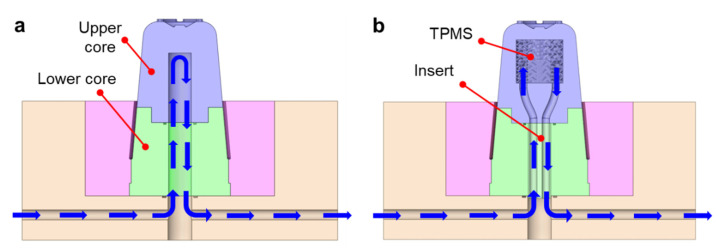
Sectional configurations of the mold cores with different cooling structures: (**a**) Baffle cooling. (**b**) TPMS cooling.

We conducted injection molding experiments to compare the cooling capability of the additively manufactured TPMS cooling structure with that of the conventional cooling channel. Figure 5a shows a sectional view of the lower mold for the conventional cooling structure, in which a baffle hole is designed through the lower and upper cores. For the TPMS-based conformal cooling, the upper core is replaced by an additively manufactured one that includes a TPMS cooling structure, as shown in Figure 5b. Here, an insert block was assembled in the baffle hole of the lower core to connect cooling circuits from the mold plate to the TPMS core. This split-type core structure was designed to reduce the manufacturing cost by reducing the proportion of additively manufactured parts.

Injection molding experiments were conducted for the two types of mold cooling structures. A hydraulic-type injection molding machine (PRO-MC 120, Dongshin Hydraulics Co. Ltd., Changwon, Korea) was used for the experiment. The polymer melt temperature was set to 230 °C, and the packing pressure was set to 50 MPa. Through the cooling channels, 50 °C water flows with a flow rate of 120 cc/s. The injection and packing times were set to 1.5 and 5.0 s, respectively. To determine the appropriate cooling time, injection molding experiments were performed by changing the cooling time from 5 to 30 s, with an increment of 5 s. The molded parts under each cooling time were measured in terms of their upper and lower diameters, with design values of 59.7 and 79.8 mm (please refer to Figure 1a).

## 3. Results and Discussion

### 3.1. Numerical Simulation of Injection Molding

**Figure 6:**
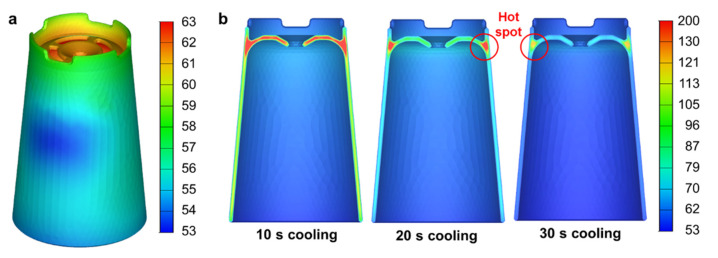
Injection molding simulation results: (**a**) Part surface temperatures at 30 s cooling time. (**b**) Change of the sectional temperature distributions.

Figure 6a shows the estimated temperature distribution of the molded part at the end of the cooling stage (i.e., 30 s cooling time). It can be seen that the temperature of the top surface is higher than that of the side surface, and the maximum surface temperature is 63°C. Although this temperature is lower than the ejection temperature of PP (100 °C), the sectional temperature distributions show hot spots at the junction regions, as shown in Figure 6b. The maximum temperatures at the hot spots are 179, 160, and 132 °C when the cooling time is 10, 20, and 30 s, respectively. This indicates that a 20 s cooling time is not enough to ensure solidification of the entire region. In addition, the cooling channel should be located near the hot spot for adaptive conformal cooling. Although the previous studies used spiral cooling channels for conformal cooling of cup-shaped parts [34,35], the spiral channels resulted in uniform mold temperature and thus are not appropriate for the adaptive cooling of hot spots. Instead, we located the TPMS cooling structure in the upper core, as shown in Figure 5b.

### 3.2. Results of Mold Heating Tests

#### 3.2.1. Polymer AM of TPMS Structures

**Figure 7:**
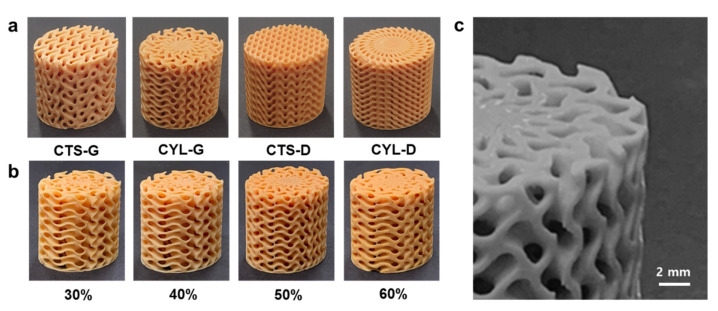
Additively manufactured TPMS structures: (**a**) Various TPMS shapes (50% volume fraction). (**b**) Various volume fractions (CYL-G type TPMS). (**c**) Microscopic image of the TPMS structure (CYL-G type TPMS with 50% volume fraction).

The designed TPMS cooling structures were fabricated by polymer AM with variations in TPMS types and base coordinates. Figure 7a shows the additively manufactured TPMS structures with different shapes (CTS-G, CYL-G, CTS-D, and CYL-D). These TPMS structures were designed to have volume fraction values within 50 ± 1%, as explained in Section 2.2. Table 4 compares the measured mass of each structure with the design value. Figure 7b shows the additively manufactured CYL-G type TPMS structures with different volume fractions, 30, 40, 50, and 60%, and the relevant mass measurement data are compared in Table 5. It can be seen that the measured mass data are close to the design values within ±3.4% deviation, which indicates that the TPMS structures were manufactured with acceptable dimensional tolerance.

Figure 7c shows a microscopic image of the CYL-G type TPMS with 50% volume fraction, which reveals that the micro-features of the TPMS structures were additively manufactured with appropriate printing resolution. Each TPMS structure was then inserted in the test mold, and the mold heating experiment was performed by circulating 80 °C hot water through the TPMS structure, as demonstrated in Figure 4b.

#### 3.2.2. Effect of the TPMS Shapes

**Figure 8:**
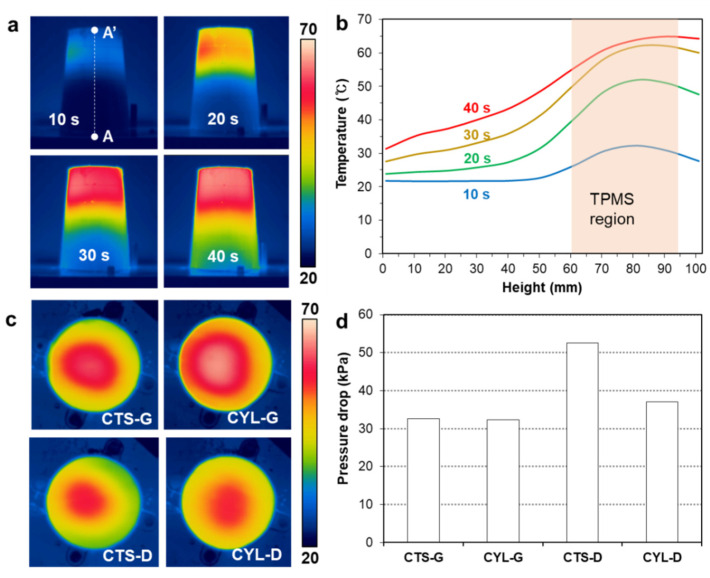
Results of heating experiments for four TPMS structures: (**a**) Change of the side temperature for the CYL-G type TPMS (unit: °C). (**b**) Temperature profiles along path A–A’. (**c**) Temperature distributions of the top surfaces at 20 s heating (unit: °C). (**d**) Comparison of pressure drops.

Figure 8a shows the temperature changes during the heating test, for the CYL-G type mold insert. It can be seen that the temperature rise is concentrated on the upper region of the mold core, which is desirable for intensively cooling the hot spots, as shown in Figure 6b. The temperature profiles along the path A–A’ are compared in Figure 8b, which demonstrates that the mold core was locally heated in the TPMS region. This localized heating capability is expected to create a localized cooling effect in the actual injection molding process in which a coolant circulates through the TPMS cooling structure. Figure 8c shows the measured temperature distributions of the top mold surfaces after 20 s heating. In terms of the TPMS shape, G-type TPMSs (CTS-G and CYL-G) show higher temperatures than D-type TPMSs (CTS-G and CYL-G). In terms of the base coordinate, TPMS structures based on the cylindrical coordinate (CYL-G and CYL-D) show higher temperatures than those based on the Cartesian coordinate (CTS-G and CTS-D).

**Figure 9:**
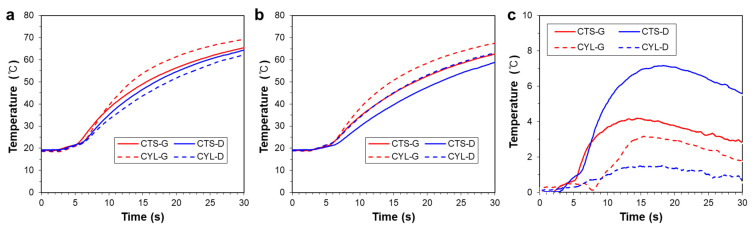
Temperature changes on the top surfaces for various TPMS shapes: (**a**) at Point 1 (P_1_); (**b**) at Point 2 (P_2_). (**c**) Temperature difference between P_1_ and P_2_.

To quantitatively investigate the temperature uniformity in the target region, temperature changes at P_1_ and P_2_ on the top surface, marked in Figure 4d, are plotted in Figure 9a,b, respectively. The corresponding temperature differences between P_1_ and P_2_ are plotted in Figure 9c, which shows that TPMSs based on the cylindrical coordinate provide more uniform temperature distributions than TPMSs based on the Cartesian coordinate.

**Figure 10:**
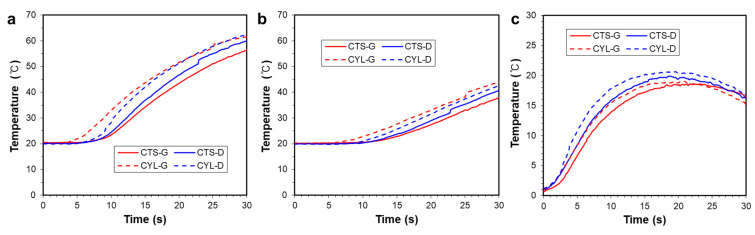
Temperature changes on the side surfaces for various TPMS shapes: (**a**) at Point 3 (P_3_); (**b**) at Point 4 (P_4_). (**c**) Temperature deviation between P_3_ and P_4_.

To quantitatively investigate the heating capability, the temperature changes at P_3_ and P_4_ on the side surface, marked in Figure 4e, are plotted in Figure 10a,b, respectively. Overall, TPMSs based on the cylindrical coordinate provide higher heating performance than TPMSs based on the Cartesian coordinate. The corresponding temperature differences, P_3_ and P_4_, are plotted in Figure 10c, showing a similar trend in which the cylindrical coordinate-based TPMS provides a larger temperature difference than the Cartesian coordinate-based TPMS. These results indicate that the TPMS based on the cylindrical coordinate ensures a superior heating performance and uniformity for a rotationally symmetric mold shape. That is, the TPMS based on the cylindrical coordinate provides a boundary conformal cellular structure [21], for a rotational symmetric geometry. The boundary conformal TPMS structure is advantageous in the flow characteristics and the resulting heat exchange capability, because an abnormally trimmed boundary region might act as a dead fluid zone [31].

Figure 8d compares the measured pressure drops between the inlet and outlet of the TPMS cooling structures. It can be seen that the gyroid type TPMSs (CTS-G and CYL-G) have a relatively low pressure drop, near 32 kPa. This pressure drop increased to 37.1 kPa for the CYL-D type TPMS, and further increased to 63.0 kPa for the CTS-D type TPMS. Therefore, the gyroid-based TPMS is advantageous in terms of fluidity (i.e., low pressure drop) compared to the diamond-based TPMS, which shows a similar trend to the previous study [30]. Based on these results, we selected the CYL-G type TPMS as the best design that provides the highest heating capability and uniformity.

#### 3.2.3. Effect of the Volume Fraction

**Figure 11:**
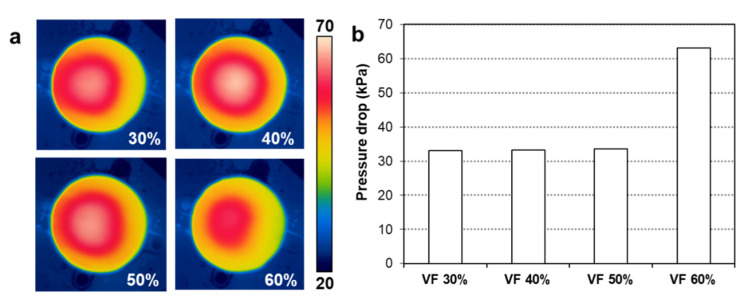
Results of heating experiments with various volume fractions: (**a**) Temperature distributions of the top surfaces at 20 s heating (unit: °C). (**b**) Comparison of pressure drops.

We conducted further investigation for the selected CYL-G type TPMS with a variation in the volume fraction. Four TPMS structures were additively manufactured with a variation in the volume fraction (VF), from 30 to 60%, as shown in Figure 7b. The mold heating tests were conducted using these TPMS inserts, and the measured temperature distributions of the top mold surfaces are compared in Figure 11a. It was observed that the 60% VF case had the lowest temperature. This can be explained by the pressure drop results of Figure 11b, in which the 60% VF case experienced a significantly higher pressure drop than the other cases. That is, a larger volume fraction of the solid part indicates a narrower fluid channel, which consequently deteriorates the fluidity of the hot water [36]. The deterioration of fluidity then reduces the heating capability of the TPMS in the heating test [37].

**Figure 12:**
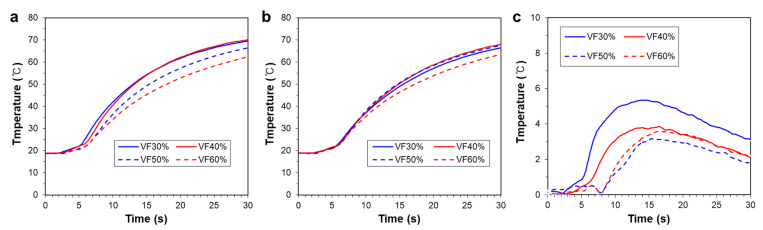
Temperature changes on the top surfaces for various volume fractions: (**a**) at Point 1 (P_1_); (**b**) at Point 2 (P_2_). (**c**) Temperature difference between P_1_ and P_2_.

To quantitatively investigate the temperature uniformity, temperature changes at P_1_ and P_2_ on the top surface are compared in Figure 12a,b, respectively. Here, the 60% VF case also has the lowest temperature, while the other three cases have higher temperatures. The corresponding temperature differences between P_1_ and P_2_ are plotted in Figure 12c, which reveals that the 50% VF case has the lowest temperature difference. Based on these results, we determined the desirable volume fraction of the CYL-G type TPMS to be 50%.

### 3.3. Results of Injection Molding Experiments

#### 3.3.1. Metal AM of the Upper Core for Injection Molding

**Figure 13:**
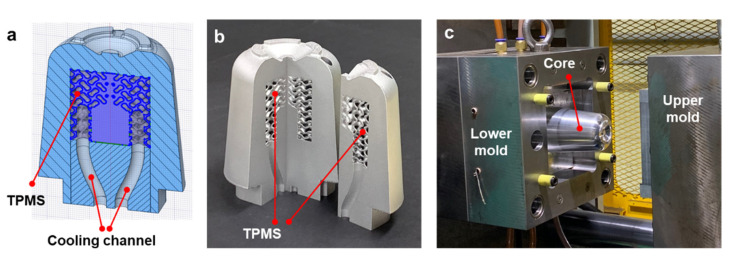
Additively manufactured metal mold containing the TPMS structure: (**a**) Sectional configuration of the upper core. (**b**) Additively manufactured upper core (sliced sample for verification of inner TPMS structure). (**c**) Experimental setup for the injection molding experiment.

With the results of the mold heating tests, we selected the CYL-G type TPMS structure with the 50% volume fraction as the best design that provides the most efficient and uniform heat transfer characteristics. The upper core part was then designed to include the CYL-G type TPMS with a 50% volume fraction. Figure 13a shows the half section of the designed core, including the TPMS and cooling channels. Figure 13b shows two sliced pieces of additively manufactured cores, which was prepared to demonstrate the inner TPMS cooling structure. The actual upper core was fabricated as a one-piece model and was assembled into the injection mold after post-processing. Figure 13c shows the experimental setup for the injection molding, where the additively manufactured TPMS core was installed.

**Figure 14:**
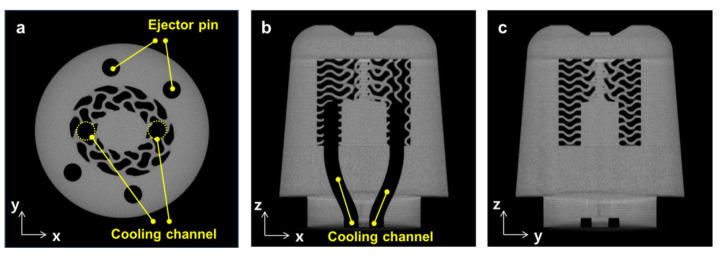
XCT images of the additively manufactured TPMS core: (**a**) XY-section. (**b**) XZ-section. (**c**) YZ-section.

To evaluate the integrity of the internal structure of the additively manufactured TPMS core, X-ray computed tomography (XCT) images were analyzed using a micro-CT scanner (Phoenix VTOMEX S240, Waygate Technologies, Wunstorf, Germany). Figure 14a shows the XCT image of the XY-section, showing that the four holes for the ejector pins and the TPMS structures with an elliptic shape are generated appropriately. Inside the TPMS domain, two holes for the vertical cooling channels are also observed. Figure 14b,c show the XCT images of the XZ- and YZ-sections, respectively. These images reveal that the microscale TPMS walls were formed appropriately, and the entire core was manufactured without a pore or defect. Accordingly, this additively manufactured mold core could be used in injection molding experiments without any occurrence of coolant leakage or dead zone [38].

#### 3.3.2. Comparison of Cooling Performance

**Figure 15:**
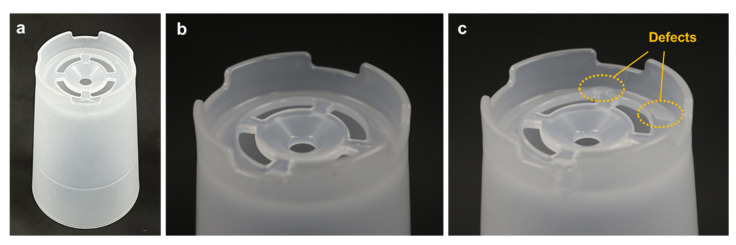
Photographs of the molded part after 10 s cooling: (**a**) TPMS conformal cooling (entire part). (**b**) TPMS conformal cooling (upper region). (**c**) Baffle cooling (upper region).

Injection molding experiments were then conducted using the two types of upper cores: the machined core with baffle cooling (Figure 5a) and the additively manufactured core with TPMS conformal cooling (Figure 13b). Both experiments were performed with increases in cooling time from 5 to 30 s. Figure 15a shows a photograph of a molded part with the TPMS conformal cooling, after 10 s cooling time. The magnified photograph of the upper region is shown in Figure 15b, in which no surface defect was found. Figure 15c shows a photograph of a molded part with the baffle cooling, which obviously reveals surface defects at the ejector-pin locations. This explains that the upper part was not fully solidified at 10 s cooling time, and thus resulted in surface defects after demolding. Accordingly, the TPMS cooling provides superior cooling performance compared to the conventional baffle cooling.

**Figure 16:**
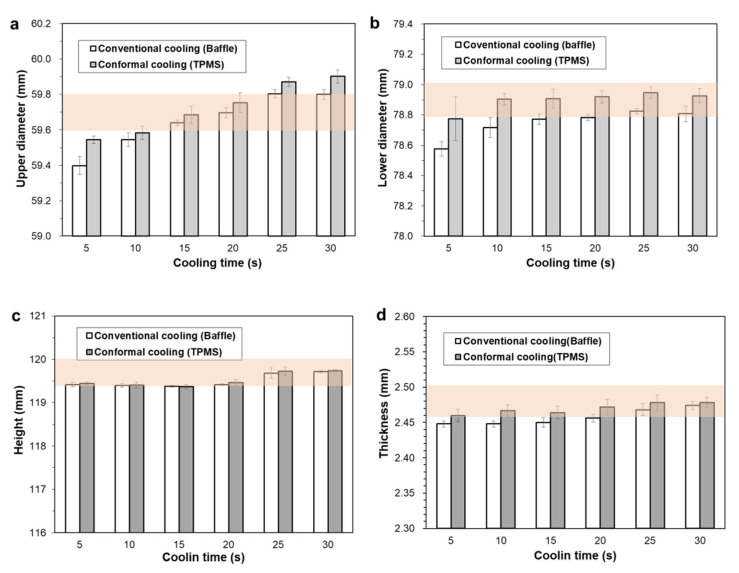
Comparison of dimensional accuracy of the molded parts according to the cooling time and method: (**a**) Upper diameter. (**b**) Lower diameter. (**c**) Cup height. (**d**) Wall thickness. In each graph, the shaded regions indicate the acceptable dimension ranges.

The cooling performances of both types of cooling were also compared in terms of the dimensional accuracy of the molded parts. Figure 16a–d show the measured dimensions of the molded parts: the upper and lower diameters, cup height, and wall thickness, respectively. Five samples were measured for every combination of cooling time and cooling method (i.e., baffle cooling and TPMS conformal cooling), and the resulting mean values and deviations are compared. In each graph, the acceptable dimensional tolerance is marked as a shaded region. The upper and lower diameters should be 59.7 ± 0.1 and 78.9 ± 0.1 mm, respectively. The acceptable dimensions for the cup height and wall thickness are 119.7 ± 0.3 and 2.48 ± 0.02 mm, respectively.

From the viewpoint of the upper diameter, both cooling methods show a similar trend in which the upper diameter increases as the cooling time increases. The minimum cooling time to meet the dimensional tolerance was then determined to be 15 s. From the viewpoint of the lower diameter, on the other hand, the TPMS conformal cooling achieved superior results over the conventional baffle cooling. In conventional cooling, the minimum cooling time is as long as 25 s to meet the dimensional tolerance. This minimum cooling time can be reduced to 10 s when conformal cooling is used. To satisfy the dimensional allowance of the cup height, the TPMS conformal cooling requires 15 s cooling time, while the conventional cooling requires 25 s cooling time. To satisfy the dimensional allowance of the wall thickness, the TPMS conformal cooling requires 10 s cooling time, while the conventional cooling requires 25 s cooling time. The appropriate cooling time that satisfies the four dimensional requirements is 15 s when the conformal cooling is used, which corresponds to a 40% reduction compared to that of the conventional cooling, 25 s. Therefore, the cooling performance of the developed mold could be improved, not only to maintain uniform mold temperature, but also to reduce part deformation, by circulating coolant through the entire microcellular region of TPMS [39].

## 4. Conclusions

In this study, we developed an adaptive conformal cooling method for injection molding by embedding a TPMS cooling structure inside a mold core. For adaptive conformal cooling, numerical simulation was conducted to predict the hot spots of the injection molded part. A mold cooling system was then designed by embedding a TPMS cooling structure near the hot spot regions. Four biomimetic TPMS structures, CTS-G, CYL-G, CTS-D, and CYL-D, were designed and fabricated using polymer AM, with combinations of different TPMS shapes and base coordinates. Through equivalent mold heating experiments, the CYL-G type TPMS was selected as the best design that provides superior heat transfer characteristics by enabling *localized-yet-uniform* temperature distribution. The mold core including the best TPMS design was fabricated using metal AM, and it was then utilized in injection molding experiments. The developed mold with the TPMS cooling structure achieved a 15 s cooling time to satisfy the dimensional tolerance of the molding parts, which corresponds to a 40% reduction of cooling time in comparison with that of the conventional mold based on baffle cooling (25 s).

In addition to the cooling time reduction, the TPMS-based conformal cooling has several advantages over the conventional channel-based conformal cooling structures, as follows:

- The TPMS-based conformal cooling enables *localized-yet-uniform* cooling, while the channel-based conformal cooling has a limitation in local cooling due to the connection of cooling channels.
- The TPMS design can be tailored to the characteristics of the target geometry for enhanced cooling performance, as we used the cylindrical base coordinate to effectively cool the mold core with a rotationally symmetric geometry.
- The TPMS conformal cooling is free from interference with the ejector pins for demolding. That is, the ejector pins can be inserted in the TPMS cooling structure, whereas the conventional conformal cooling channels must be designed to avoid interference with ejector pins.

Based on these advantages, the proposed TPMS cooling structure is expected to be effectively used for adaptive conformal cooling of injection molds when there is a hot spot to be cooled intensively. Moreover, the proposed adaptive conformal cooling method can be efficiently used in the injection molding of large parts by inserting a number of TPMS cooling structures near hot spot regions. This approach is cost-effective because it minimizes the volume of additively manufactured parts, of which costs are much higher than those of the conventional machined parts.

## Figures and Tables

**Table 1:** Thermal and mechanical properties of used materials.

Material	AISI-1055	AA7075	AlSi7Mg
Density (kg/m^3^)	7850	2810	2680
Specific heat (J/kg-K)	486	960	963
Thermal conductivity (W/m-K)	46.9	173	167
Thermal expansion (μm/m-K)	11.9	23.0	21.4
Elastic modulus (GPa)	206	71.7	72.4
Yield strength (MPa)	392	103	107

**Table 2:** Comparison of TPMS structures with different combinations of unit cells and base coordinates.

TPMS Type	CTS-G	CYL-G	CTS-D	CYL-D
Unit cell	Gyroid (G)	Gyroid (G)	Diamond (D)	Diamond (D)
Base grid	Cartesian	Cylindrical	Cartesian	Cylindrical
Volume fraction	50.7%	49.3%	50.7%	49.5%
Surface area (mm^2^)	28,865	29,894	34,747	34,563

**Table 3:** Injection molding conditions for numerical simulation.

Molding Condition	Value	Molding Condition	Value
Filling time (s)	6.5	Injection pressure (MPa)	50
Packing time (s)	3.0	Injection speed (cc/s)	11.1
Cooling time (s)	30	Packing pressure (MPa)	40
Melt temperature (°C)	200	Coolant temperature (°C)	60

**Table 4:** Comparison for the mass of various TPMS shapes (volume fraction: 50%).

TPMS Shape		CTS-G	CYL-G	CTS-D	CYL-D
Volume (mm^3^)		16,470.7	16,991.0	16,470.5	17,383.3
Mass (g)	Designed	21.92	22.61	21.92	23.14
	Measured	21.51	22.72	21.20	23.09
	Error	−1.90%	0.48%	−3.39%	−0.22%

**Table 5:** Comparison for the mass of CLY-G type TPMSs with different volume fractions.

Volume Fraction		30%	40%	50%	60%
Volume (mm^3^)		9998.9	13,475.1	16,991.0	20,249.8
Mass (g)	Designed	13.31	17.93	22.61	26.95
	Measured	13.66	18.17	22.72	26.37
	Error	2.56%	1.32%	0.48%	−2.20%
